# Supplementary material for: Predicting the evolution of the Lassa virus endemic area and population at risk over the next decades
Source: Nat Commun. 2022 Sep 27;13:5596. doi: 10.1038/s41467-022-33112-3 (PMC9515147; doi:10.1038/s41467-022-33112-3)
Supplement: Supplementary file 1 — Supplementary Information [file 41467_2022_33112_MOESM1_ESM.pdf]

Predicting the evolution of the Lassa virus endemic area and population at risk over the next decades – Supplementary Files (Figures S1-S14, Tables S1-S6)

Raphaëlle Klitting[1], Liana E. Kafetzopoulou[2,3], Wim Thiery[4], Gytis Dudas[5], Sophie Gryseels[6,7], Anjali Kotamarthi[1], Bram Vrancken[2], Karthik Gangavarapu[1], Mambu Momoh[8,9], John Demby Sandi[9], Augustine Goba[9], Foday Alhasan[9], Donald S. Grant[9,10], Sylvanus Okogbenin[11,12], Ephraim Ogbaini-Emovo[11], Robert F. Garry[13,14,15], Allison R. Smither[13], Mark Zeller[1], Matthias G. Pauthner[1], Michelle McGraw[1], Laura D. Hughes[16], Sophie Duraffour[3,17], Stephan Günther[3,17], Marc A. Suchard[18,19,20], Philippe Lemey[2], Kristian G. Andersen[1,21], Simon Dellicour[2,22]

[1] Department of Immunology and Microbiology, The Scripps Research Institute, La Jolla, CA 92037, USA

[2] Department of Microbiology, Immunology and Transplantation, Rega Institute, Laboratory for Clinical and Epidemiological Virology, KU Leuven - University of Leuven, Leuven, Belgium

[3] Bernhard Nocht Institute for Tropical Medicine, Hamburg, Germany

[4] Department of Hydrology and Hydraulic Engineering, Vrije Universiteit Brussel, Brussels, Belgium

[5] Institute of Biotechnology, Life Sciences Center, Vilnius University, Vilnius, Lithuania

[6] Evolutionary Ecology group, Department of Biology, University of Antwerp, 2610 Antwerp, Belgium

[7] Vertebrate group, Directorate Taxonomy and Phylogeny, Royal Belgian Institute of Natural Sciences, 1000 Brussels, Belgium

[8] Eastern Technical University of Sierra Leone, Sierra Leone

[9] Viral Hemorrhagic Fever Program, Kenema Government Hospital, Ministry of Health and Sanitation, Sierra Leone

[10] College of Medicine and Allied Health Sciences, University of Sierra Leone

[11] Irrua Specialist Teaching Hospital, Irrua, Nigeria

[12] Faculty of Clinical Sciences, College of Medicine, Ambrose Alli University, Ekpoma, Nigeria

[13] Department of Microbiology and Immunology, Tulane University, School of Medicine, New Orleans, LA, 70112 USA

[14] Zalgen Labs, LCC, Frederick, MD 21703, USA

[15] Global Virus Network (GVN), Baltimore, MD 21201, USA

[16] Department of Integrative, Structural and Computational Biology, The Scripps Research Institute, La Jolla, CA 92037 USA

[17] German Center for Infection Research (DZIF), Partner site Hamburg–Lübeck–Borstel–Riems, Hamburg, Germany

[18] Department of Biomathematics, David Geffen School of Medicine, University of California, Los Angeles, CA, USA

[19] Department of Biostatistics, Fielding School of Public Health, University of California, Los Angeles, CA, USA

[20] Department of Human Genetics, David Geffen School of Medicine, University of California, Los Angeles, CA, USA

[21] Scripps Research Translational Institute, La Jolla, CA 92037, USA

[22] Spatial Epidemiology Lab (SpELL), Université Libre de Bruxelles, CP160/12 50, av. FD Roosevelt, 1050 Bruxelles, Belgium

Corresponding authors: Raphaëlle Klitting (rklitting@scripps.edu), Simon Dellicour (simon.dellicour@ulb.be)

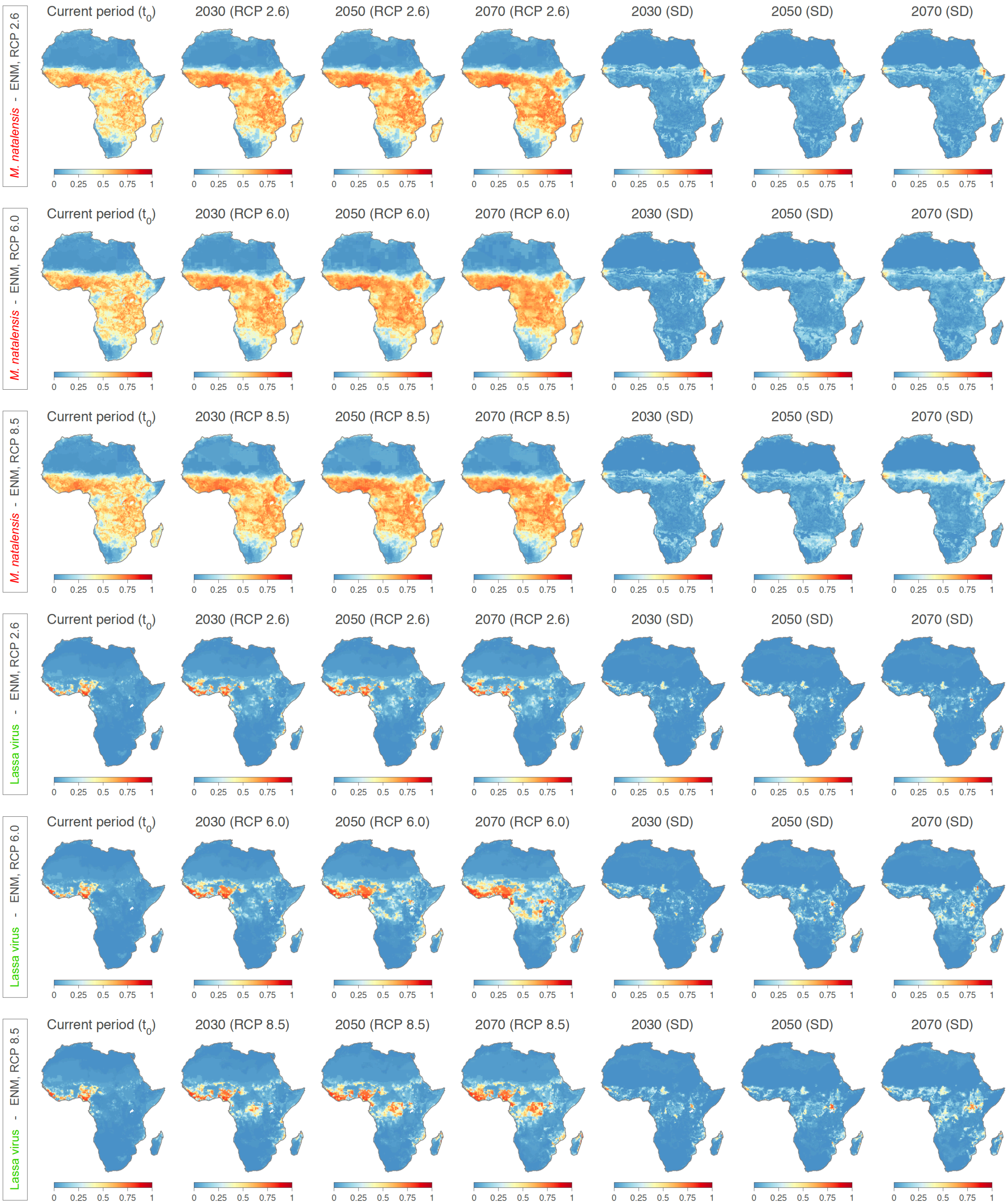

Figure S1. Predicted ecological niche suitability for *Mastomys natalensis* and Lassa virus for the current period, 2030, 2050, and 2070, as well as associated standard deviation (SD) between climatic models. Each future projection (i.e. for 2030, 2050, and 2070) was performed according to four different climatic models and three different representative concentration pathways (RCPs), i.e. greenhouse gas concentration scenarios defined by the Intergovernmental Panel on Climate Change (IPCC): RCP 2.6, RCP 6.0, and RCP 8.5. For a specific RCP and time period, we here report predicted probabilities averaged over the projections obtained with the four different climatic models (see the text for further detail). We also report the SD between the projections obtained for the four climatic models.

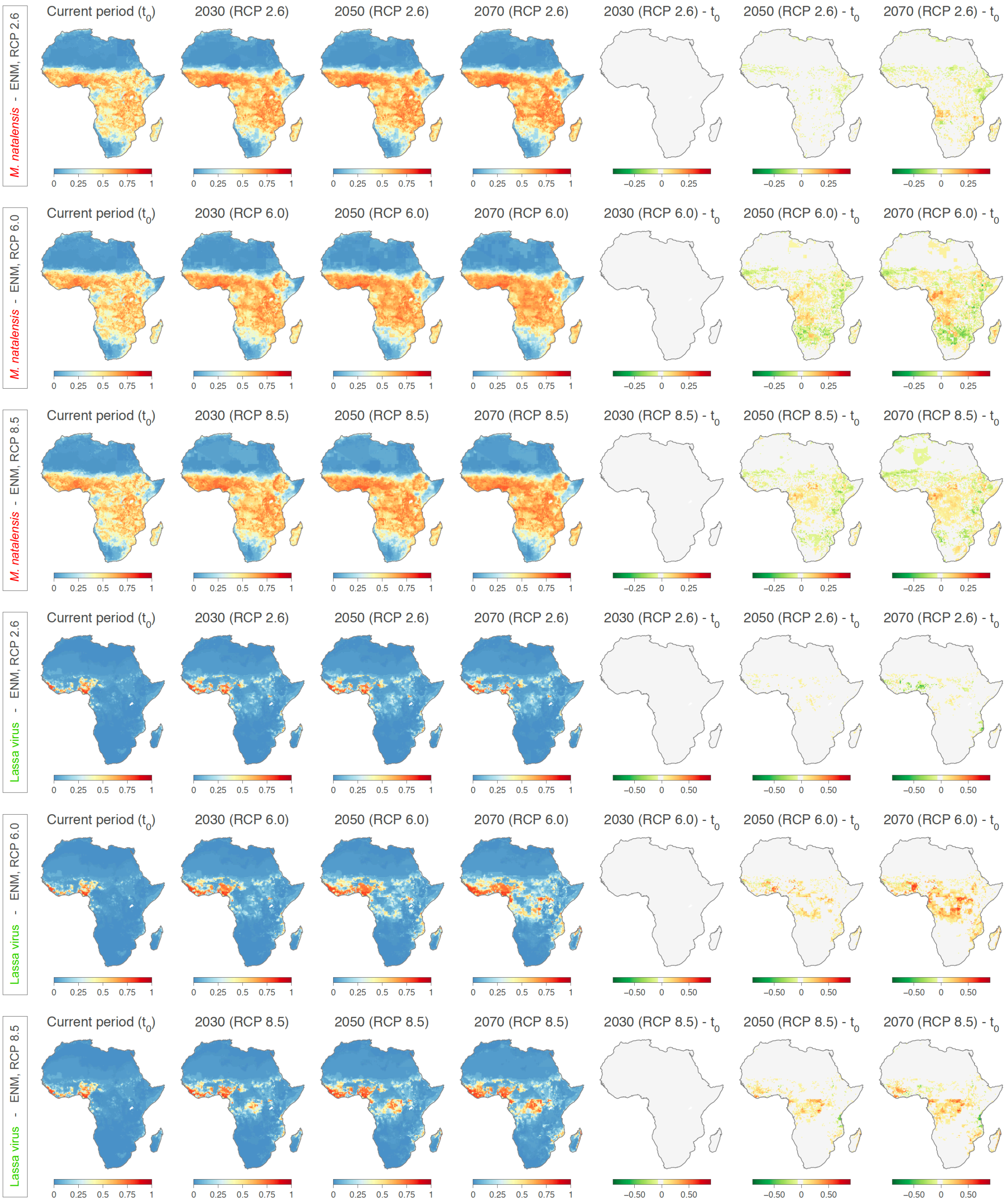

Figure S2. Predicted ecological niche suitability for *Mastomys natalensis* and Lassa virus for the current period, 2030, 2050, and 2070, as well as differences between future and current projections. Each future projection (i.e. for 2030, 2050, and 2070) was performed according to four different climatic models and three different representative concentration pathways (RCPs), i.e. greenhouse gas concentration scenarios defined by the Intergovernmental Panel on Climate Change (IPCC): RCP 2.6, RCP 6.0, and RCP 8.5 (IPCC scenario also commonly referred as "business as usual"). For a specific RCP and time period, we here report predicted probabilities averaged over the projections obtained with the four different climatic models (see the text for further detail). We also report the difference between each future projection and the projection obtained for the current period (t_0).

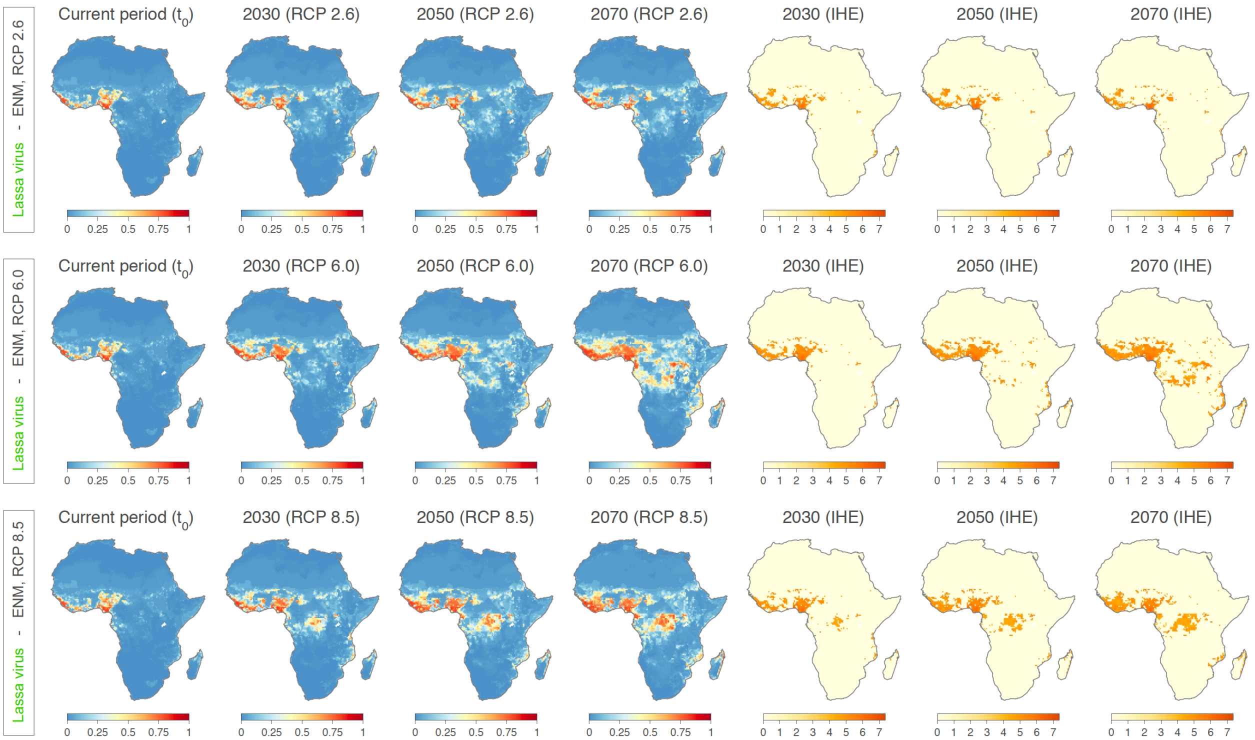

Figure S3. Predicted ecological niche suitability of Lassa virus for the current period, 2030, 2050, and 2070, as well as associated estimations for an index of human exposure (IHE). Each future projection (i.e. for 2030, 2050, and 2070) was performed according to three different representative concentration pathways (RCPs) and averaged over projections obtained according to four climatic models (see the text for further detail). IHE were obtained by only reporting human population estimates ($\log_{10}$-transformed) associated with Lassa virus predicted probability higher than 0.5.

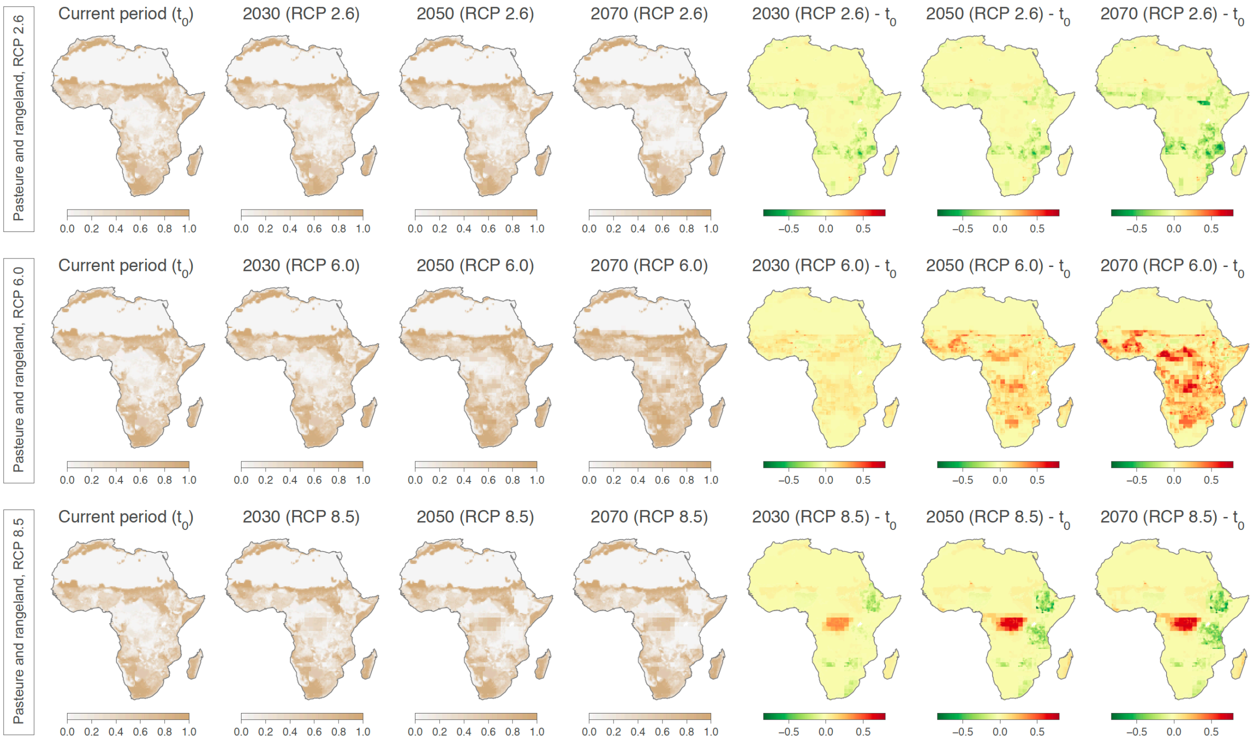

Figure S4. Current and future estimates for the "pastures and rangeland" environmental factor used in the ENM analyses. We here also report the difference between each future estimate and the estimate for the current period (t_0).

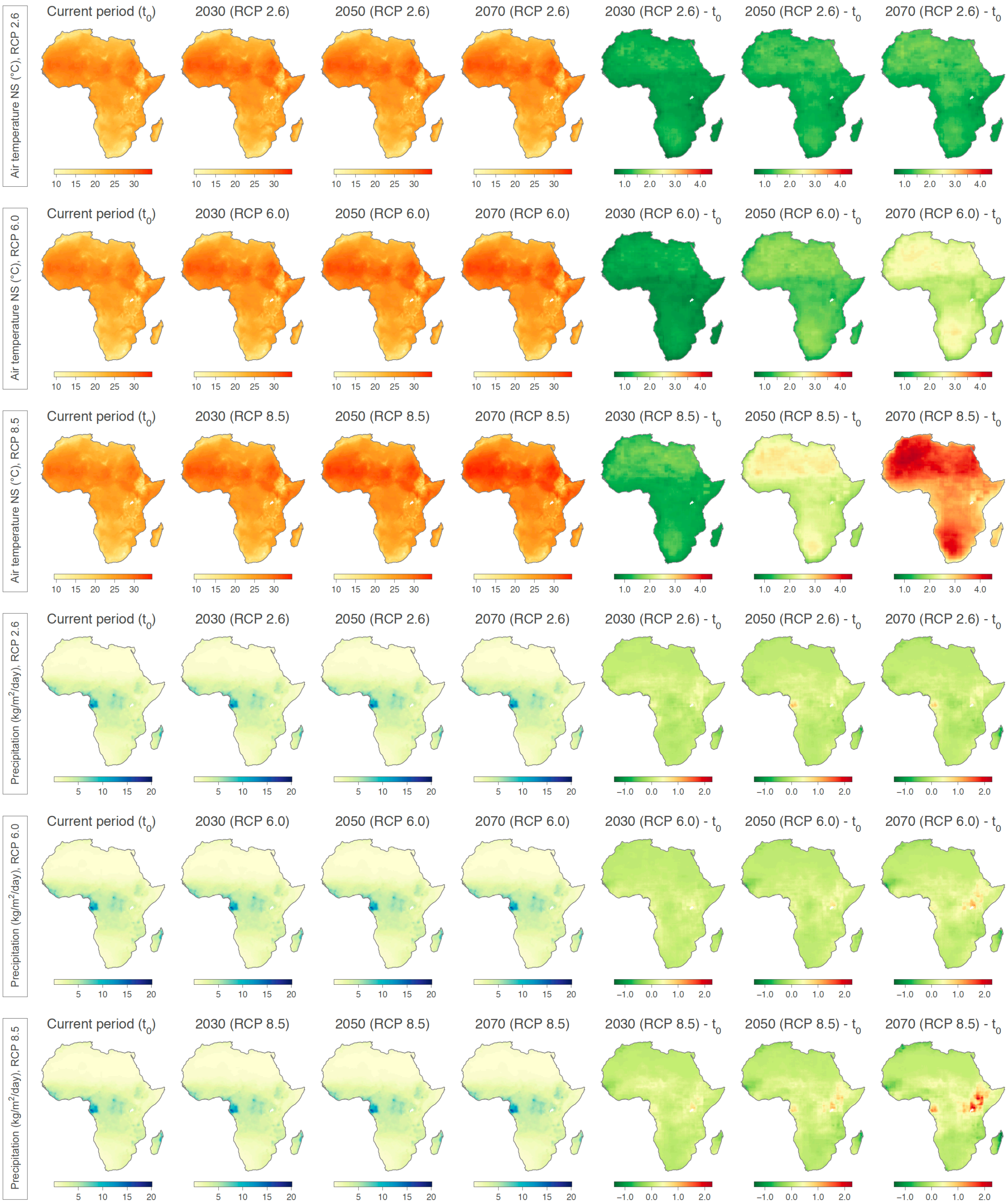

Figure S5. Current and future estimates for the two climatic factors used in the ENM analyses: the air temperature near the surface (NS) and the precipitation rate. We here also report the difference between each future estimate and the estimate for the current period (t_0).

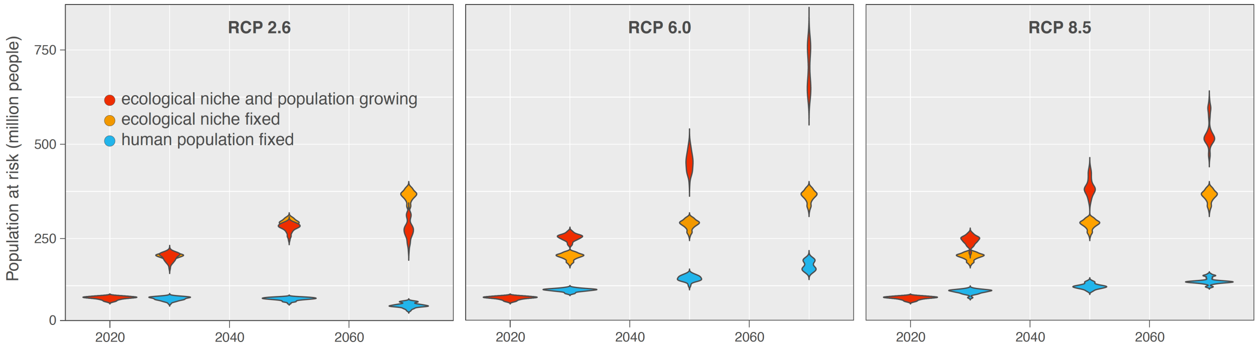

Figure S6. Projections of the human population at risk of exposure to Lassa virus. Each future projection (i.e. for 2030, 2050, and 2070) was obtained according to three different representative concentration pathways (RCPs) and averaged over projections obtained according to four climatic models (see the text for further detail). In addition, we also re-estimate these projections while fixing the human population, i.e. not using the future projections of human population to estimate the number of people at risk. Source data are provided as a Source Data file.

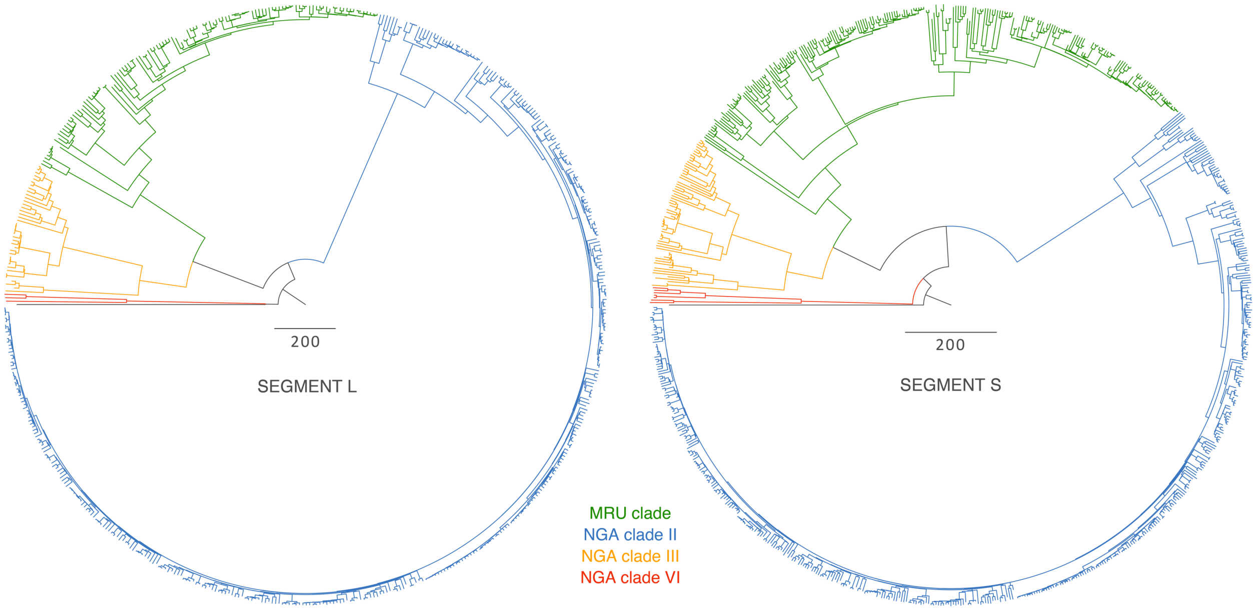

Figure S7. Overall Lassa virus time-scaled phylogenetic trees based on segments L and S. Clades are coloured according to the main Lassa virus clades.

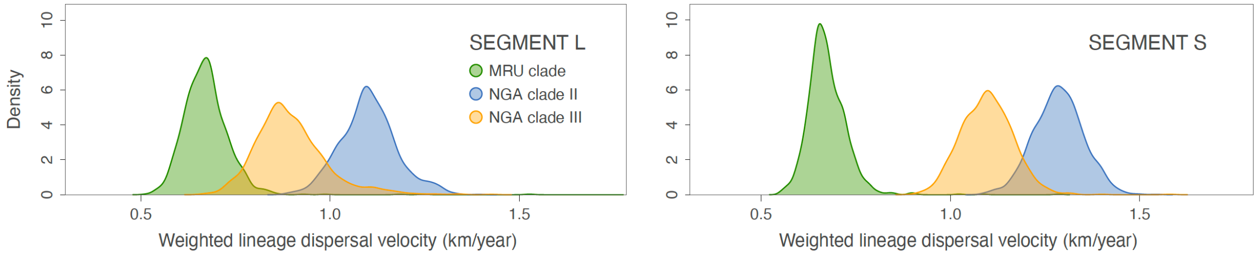

Figure S8. Weighted dispersal velocities estimated for Lassa virus lineages. These estimations are based on 1,000 posterior trees obtained by continuous phylogeographic inference.

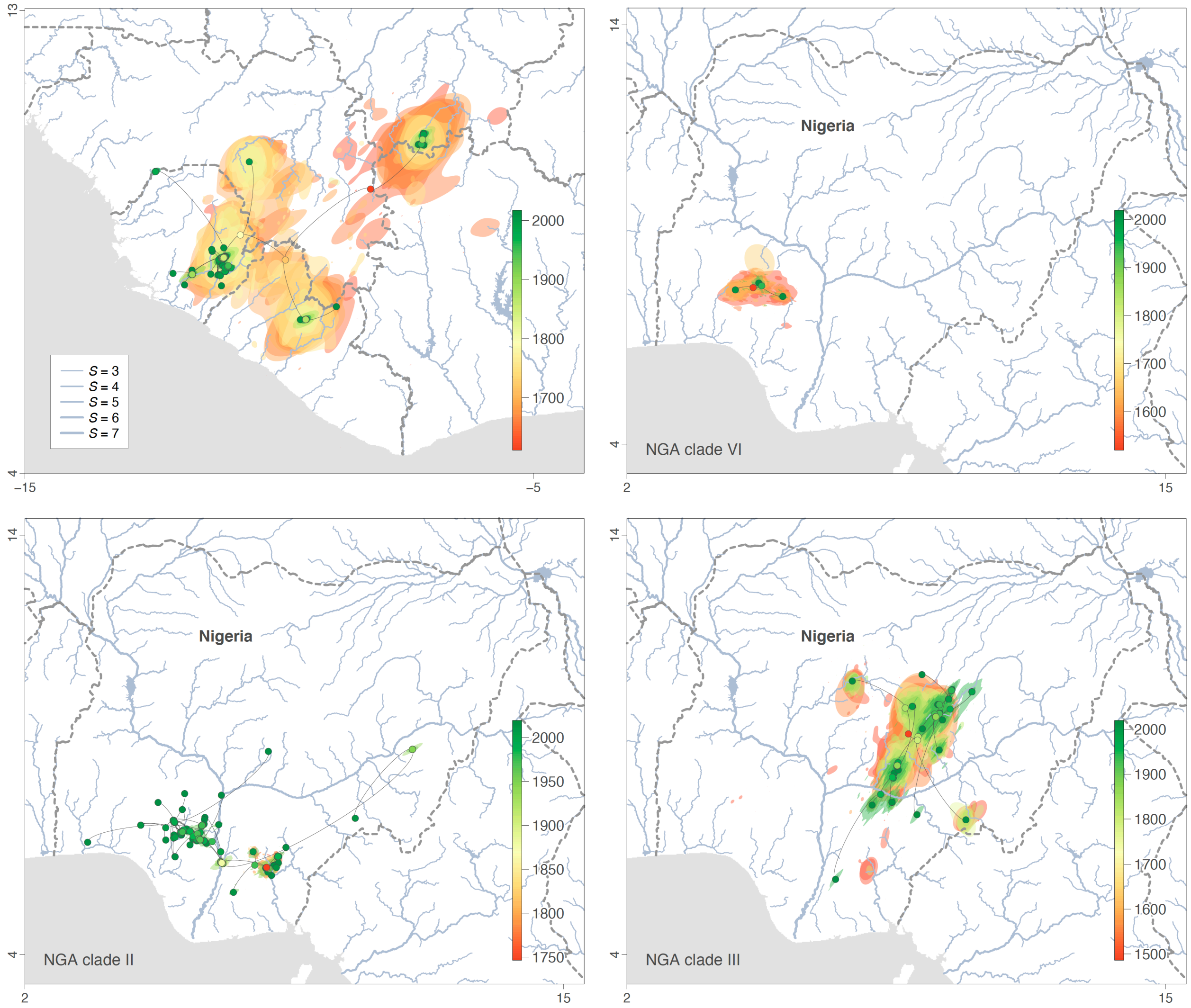

Figure S9. Spatiotemporal diffusion of Lassa virus lineages in western Africa and Nigeria, based on the analysis of segment L. MCC (maximum clade credibility) tree obtained by continuous phylogeographic inference based on 1,000 posterior trees. A distinct phylogeographic analysis has been based on segments L and S as well as, in the case of the Nigerian data set, on clades VI, II and III. These MCC trees are superimposed on 80% HPD reflecting phylogeographic uncertainty. Nodes of the trees, as well as HPD regions, are coloured from red (the time to the most recent common ancestor, TMRCA) to green (most recent sampling time), and oldest nodes (and corresponding HPD regions) are here plotted on top of youngest nodes. The trees are superimposed on maps displaying the main rivers present in the study area and classified according to their Strahler number *S*, which measures the importance of a river by counting the number of upstream rivers connected to it. International borders are represented by white dashed lines.

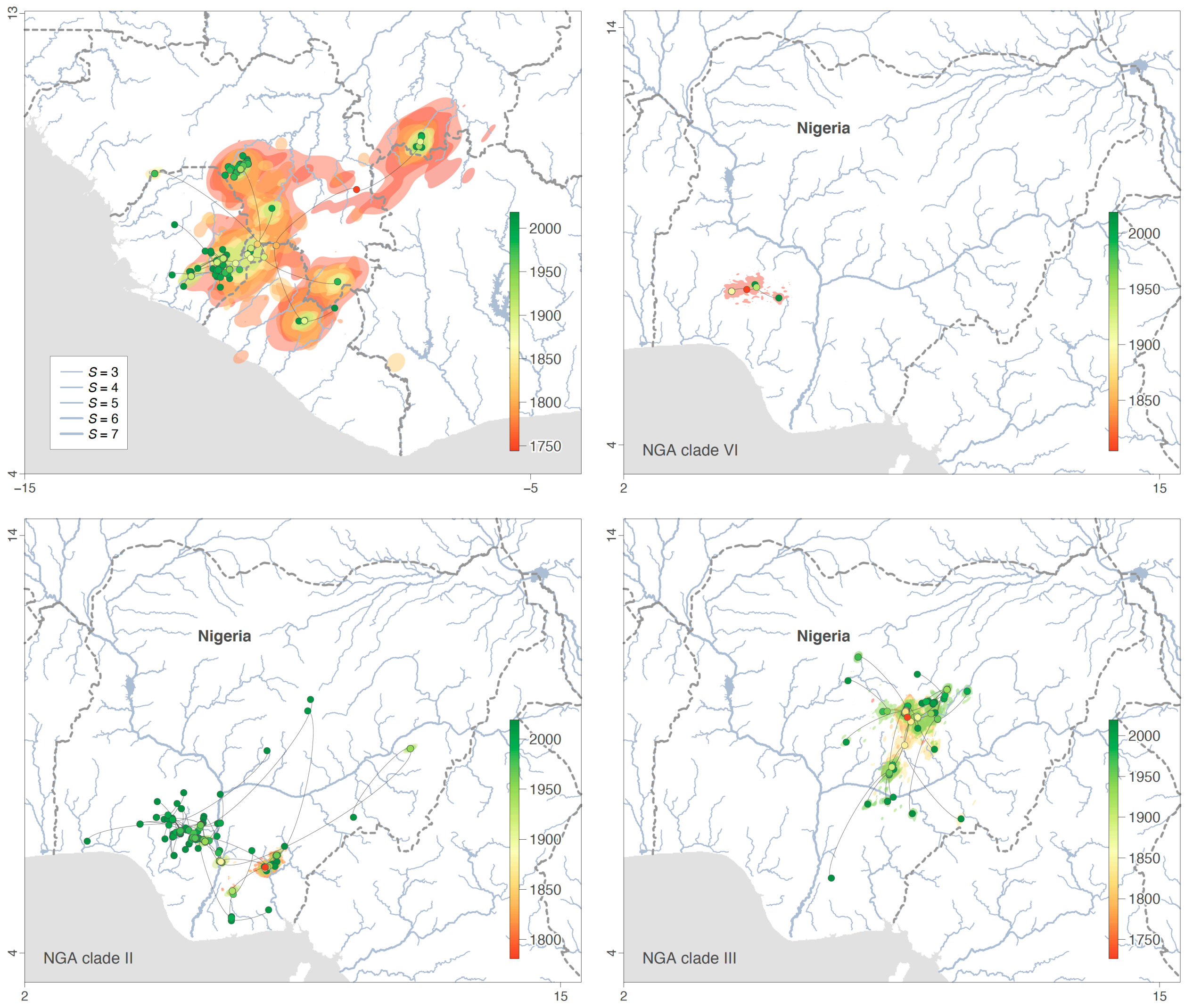

Figure S10. Spatiotemporal diffusion of Lassa virus lineages in western Africa region and Nigeria, based on the analysis of segment S. MCC (maximum clade credibility) tree obtained by continuous phylogeographic inference based on 1,000 posterior trees. A distinct phylogeographic analysis has been based on segments L and S as well as, in the case of the Nigerian data set, on clades VI, II and III. These MCC trees are superimposed on 80% HPD reflecting phylogeographic uncertainty. Nodes of the trees, as well as HPD regions, are coloured from red (the time to the most recent common ancestor, TMRCA) to green (most recent sampling time), and oldest nodes (and corresponding HPD regions) are here plotted on top of youngest nodes. The trees are superimposed on maps displaying the main rivers present in the study area and classified according to their Strahler number S, which measures the importance of a river by counting the number of upstream rivers connected to it. International borders are represented by white dashed lines.

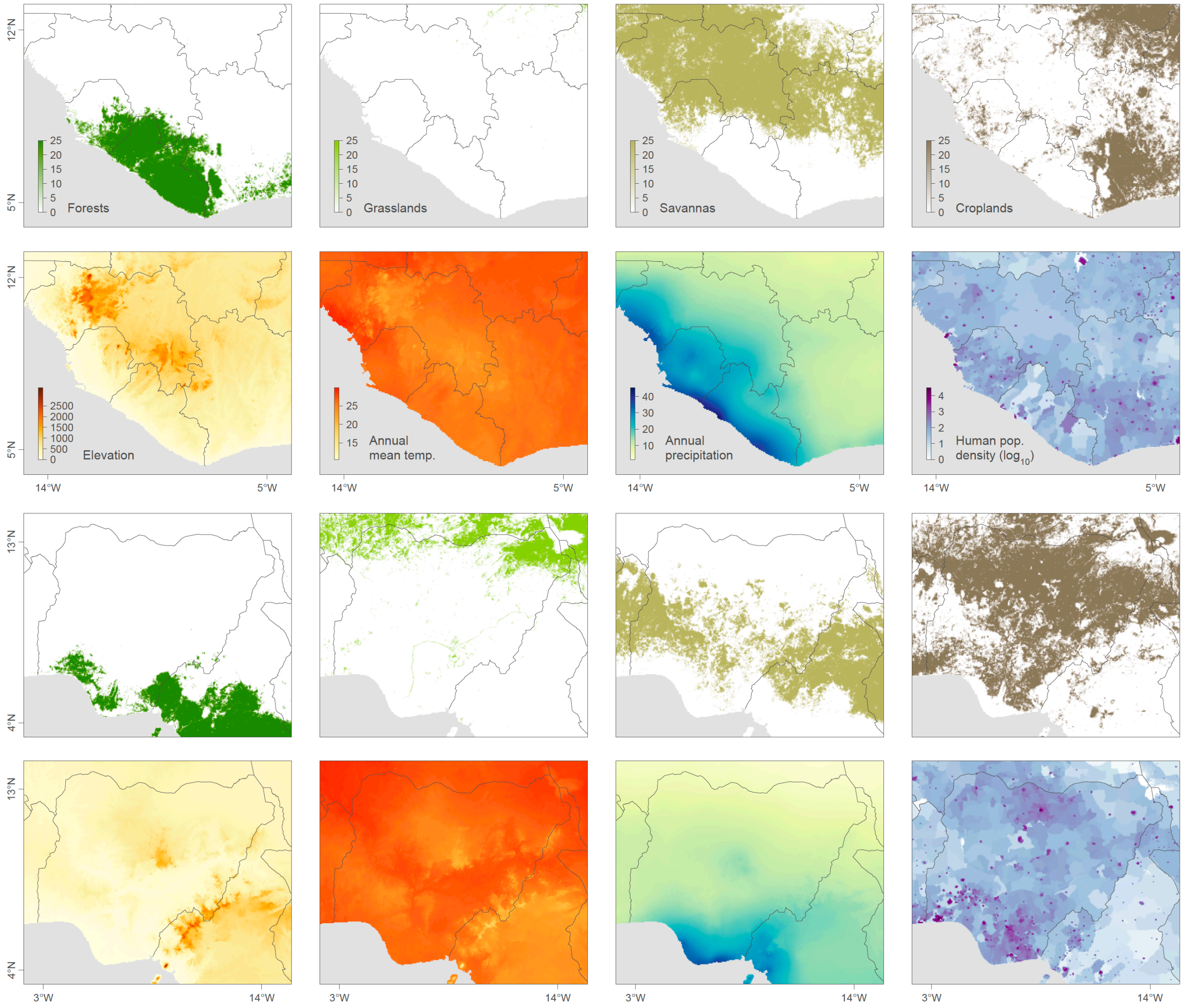

Figure S11. Environmental variables tested for their impact on the dispersal velocity of Lassa virus lineages in western Africa and in Nigeria. Elevation is reported in meters, mean annual temperature is reported in Celsius degrees, and annual precipitation is reported in meters per year.

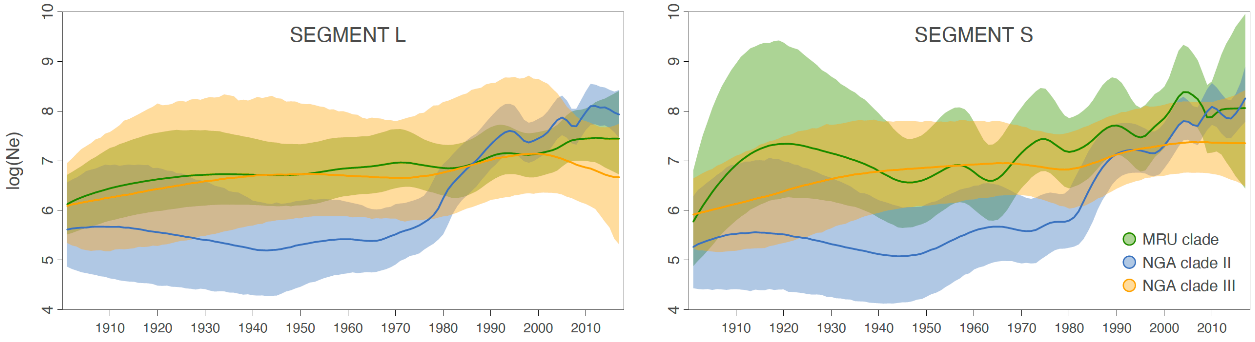

Figure S12. Estimation of the viral effective population through time. We here report Bayesian skygrid effective population size estimates based on the analysis of both segments (L and S) and for all main Lassa virus clades. Solid curves and surrounding shaded polygons correspond to median estimates and 95% HPD region, respectively.

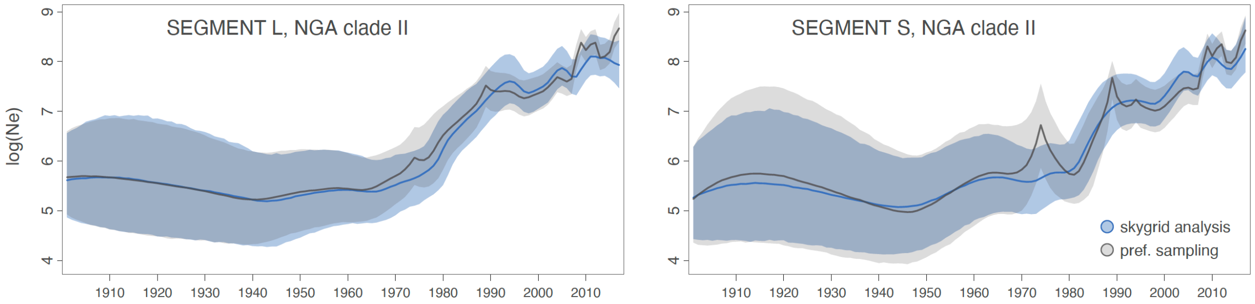

Figure S13. Estimation of the viral effective population through time for NGA clade II. We here report Bayesian skygrid effective population size estimates based on the analysis of both segments (L and S) for NGA clade II (in blue), as well as the results of the corresponding preferential sampling analysis (in grey). Contrary to the skygrid analysis, the preferential sampling analysis accounts for heterogeneous sampling density through time, which can improve estimates of global effective population size. In the graphs, solid curves and surrounding shaded polygons correspond to median estimates and 95% HPD region, respectively. See Figure S12 for a comparison with the skygrid reconstructions obtained for the MRU clade and NGA clade III.

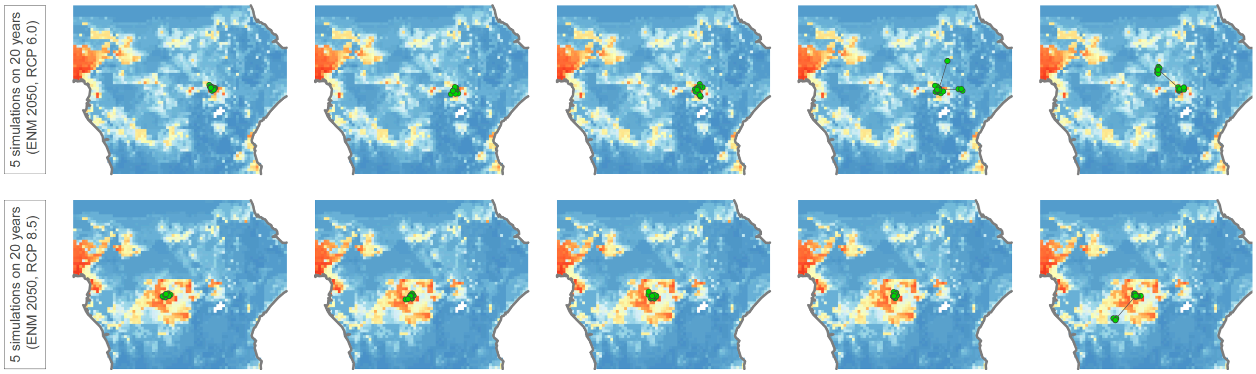

Figure S14. Phylogeographic simulations of a viral spread following a successful introduction into a new ecologically suitable area. Phylogeographic simulations are based on the tree topologies inferred for Lassa virus clade II (segment S). Five out of 1,000 simulated trees are shown superimposed on maps displaying future ecological suitability estimated for Lassa virus. All 1,000 trees were simulated starting from the same ancestral location and virus dispersal was constrained by ecological suitability using ecological niche projections for Lassa virus in 2050 according to scenarios RCP 6.0 (upper panels) and RCP 8.5 (lower panels).

Table S1. Evolution of the population living in areas ecologically suitable for Lassa virus. For each scenario (RCP 2.6, 6.0, and 8.5), time period (2030, 2050, and 2070), and considered cut-off value of ecological suitability (0.25, 0.50, and 0.75), we calculated the number of people at risk of exposure to Lassa virus (in millions) from index of human exposure (IHE) values (projected). To estimate the effect of the increase in population living in current suitable areas we combined current projected ecologically suitable areas with future population projections to re-estimate IHE values. To estimate the effect of population growth, we used current population projections combined with future projected ecologically suitable areas and re-estimated IHE values (current pop.). For all population estimates, we provide the mean, and 95% HPD interval across estimates obtained with all climatic models and ecological niche model replicates. Counts for endemic areas correspond to the sum of the counts for Guinea, Liberia, Nigeria and Sierra Leone, counts for Africa are for the whole African continent. Here, values are only shown for the intermediate scenario, i.e. RCP 6.0. Source data are provided for all scenarios as a Source Data file.

		t_0	2030			2050			2070		
**Cut-off**	**Location**	**projected**	**projected**	**current area**	**current pop.**	**projected**	**current area**	**current pop.**	**projected**	**current area**	**current pop.**
**0.25**	**Guinea**	4<br>[3-4]	11<br>[10-11]	7<br>[6-7]	5<br>[5-6]	13<br>[13-14]	8<br>[7-8]	6<br>[6-6]	14<br>[14-15]	8<br>[8-9]	6<br>[6-6]
	**Liberia**	1<br>[1-1]	7<br>[6-7]	4<br>[3-4]	2<br>[2-2]	10<br>[9-10]	6<br>[5-6]	2<br>[2-2]	12<br>[12-12]	7<br>[6-7]	2<br>[2-2]
	**Nigeria**	89<br>[87-90]	177<br>[175-179]	204<br>[201-207]	79<br>[78-80]	267<br>[261-271]	296<br>[292-300]	82<br>[81-83]	357<br>[350-368]	380<br>[375-385]	85<br>[84-88]
	**Sierra Leone**	3<br>[3v3]	7<br>[7-7]	7<br>[7-7]	3<br>[3-3]	10<br>[10-10]	9<br>[9-9]	3<br>[3-3]	11<br>[11-11]	10<br>[10-10]	3<br>[3-3]
	**Endemic areas**	97<br>[95-98]	202<br>[199-205]	222<br>[218-225]	89<br>[88-91]	300<br>[292-304]	319<br>[313-323]	94<br>[92-95]	394<br>[387-406]	405<br>[398-411]	97<br>[95-99]
	**Africa**	124<br>[123-125]	368<br>[358-383]	273<br>[269-274]	162<br>[157-168]	677<br>[624-737]	384<br>[379-386]	215<br>[202-231]	1011<br>[933-1091]	480<br>[474-483]	260<br>[241-280]
**0.50**	**Guinea**	2<br>[2-2]	8<br>[7-8]	4<br>[4-5]	4<br>[4-4]	10<br>[9-11]	5<br>[4-5]	4<br>[4-5]	12<br>[11-13]	5<br>[5-6]	5<br>[5-5]
	**Liberia**	1<br>[1-1]	5<br>[4-5]	3<br>[3-3]	2<br>[2-2]	7<br>[6-9]	4<br>[4-4]	2<br>[2-2]	11<br>[10-12]	5<br>[5-5]	2<br>[2-2]
	**Nigeria**	76<br>[70-80]	167<br>[154-172]	173<br>[159-182]	74<br>[69-76]	255<br>[247-259]	251<br>[231-265]	79<br>[76-80]	335<br>[328-341]	321<br>[296-339]	81<br>[79-82]
	**Sierra Leone**	3<br>[3-3]	7<br>[7-7]	7<br>[7-7]	3<br>[3-3]	9<br>[9-9]	8<br>[8-9]	3<br>[3-3]	11<br>[11-11]	10<br>[10-10]	3<br>[3-3]
	**Endemic areas**	83<br>[76-87]	187<br>[172-192]	186<br>[172-196]	83<br>[77-86]	282<br>[271-288]	268<br>[248-283]	88<br>[85-90]	368<br>[359-377]	341<br>[315-359]	91<br>[89-93]
	**Africa**	92<br>[83-98]	253<br>[233-268]	203<br>[186-214]	113<br>[104-120]	453<br>[414-497]	288<br>[264-304]	144<br>[128-157]	700<br>[624-779]	363<br>[333-384]	179<br>[159-199]
**0.75**	**Guinea**	1<br>[1-2]	5<br>[4-6]	2<br>[2-3]	3<br>[2-3]	7<br>[5-8]	3<br>[3-3]	3<br>[2-3]	8<br>[6-9]	3<br>[3-4]	3<br>[2-4]
	**Liberia**	1<br>[1-1]	3<br>[3-4]	2<br>[1-2]	1<br>[1-1]	6<br>[5-6]	3<br>[2-3]	2<br>[1-2]	8<br>[8-10]	3<br>[2-4]	2<br>[2-2]
	**Nigeria**	45<br>[39-50]	120<br>[93-135]	98<br>[86-110]	54<br>[42-61]	198<br>[160-221]	142<br>[124-159]	61<br>[49 68]	259<br>[195-295]	182<br>[159-204]	63<br>[47-71]
	**Sierra Leone**	3<br>[3-3]	7<br>[7-7]	7<br>[7-7]	3<br>[3-3]	9<br>[9-9]	8<br>[8-8]	3<br>[3-3]	11<br>[10-11]	10<br>[10-10]	3<br>[3-3]
	**Endemic areas**	50<br>[44-55]	135<br>[107-152]	109<br>[96-122]	61<br>[48-68]	219<br>[179-245]	156<br>[137-174]	69<br>[56-77]	286<br>[220-325]	197<br>[174-221]	71<br>[54-80]
	**Africa**	51<br>[45-57]	154<br>[118-175]	112<br>[98-125]	69<br>[54-79]	268<br>[207-317]	159<br>[139-179]	85<br>[68-99]	393<br>[288-466]	201<br>[175-226]	100<br>[72-117]

Table S2. Impact of main rivers on the dispersal frequency of Lassa virus lineages. The results are based on 100 posterior trees obtained by spatially-explicit phylogeographic inference. For each category of rivers, we report the total cost (TC, i.e. the sum of the least-cost values computed for each phylogenetic branch considered separately) and associated Bayes factor (BF) support indicating if TC is lower than expected by chance, i.e. lower than the value computed in the null dispersal model. Following Kass & Raftery (1995), we consider a BF value >20 as strong support for a significant correlation between the environmental distances and dispersal durations. A river is here characterised by its Strahler number (*S*), which measures its importance by looking at the number of upstream rivers connected to it. *k* is the rescaling parameter used to transform the initial raster and defines the additional resistance to movement when the cell does contain such a potential landscape barrier (see the Methods section for further detail). For each estimated TC value, we report both the median estimate and the 95% HPD interval, as well as the associated Bayes factor (BF) support. (*) Analyses based on main rivers associated with a *S* higher than five were only performed for lineages occurring in Nigeria.

Rivers category	k	Segment L		Segment S	
		TC	BF	*TC*	BF
***S* > 2**	10	11692 [11315-12173]	3.0	6930 [6356-7512]	3.0
	100	15842 [14937-16890]	1.8	9260 [8349-9800]	2.0
	1000	32868 [27776-39707]	1.8	19167 [15167-22463]	1.6
***S* > 3**	10	11258 [10881-11722]	3.2	6674 [6124-7251]	3.2
	100	13230 [12530-14010]	3.0	7786 [7058-8352]	2.4
	1000	27414 [23237-33115]	1.0	16084 [12827-19439]	1.1
***S* > 4**	10	11076 [10699-11551]	3.3	6573 [6032-7157]	3.3
	100	11962 [11398-12618]	4.9	6997 [6313-7652]	2.8
	1000	18723 [16556-23418]	4.0	10851 [8604-12816]	1.1
S* > 5	10	106662 [103866-108759]	3.2	57034 [55872-58355]	3.2
	100	125682 [122927-128087]	3.5	66919 [65547-68244]	1.6
	1000	235351 [228537-242152]	3.3	122884 [119306-125924]	0.7
S* > 6	10	106076 [103352-108162]	3.2	56758 [55570-58068]	3.0
	100	122850 [120201-125005]	3.3	65324 [64124-66637]	1.5
	1000	193963 [189229-199856]	1.4	101658 [99346-103853]	0.4

Table S3. Impact of several environmental factors on the dispersal velocity of Lassa virus tip branches, based on the analysis of segment L. The results are based on 100 posterior trees obtained by spatially-explicit phylogeographic inference. For the main rivers *S* refers to the Strahler number, which measures the importance of a river by counting the number of upstream rivers connected to it. "C" and "R" indicate if the considered environmental raster was considered as a conductance ("C") or resistance factor ("R"), "*k*" is the rescaling parameter used to transform the initial raster, and "*S*" refers to the Strahler number that measures its importance by looking at the number of upstream rivers connected to it (see the text for further detail). For regression coefficients and *Q* values we report both the median estimate and the 95% HPD interval. The Bayes factor (BF) supports are only reported when p(*Q* > 0) is at least 90%. Following Kass & Raftery (1995), we consider a BF value >20 as strong support for a significant correlation between the environmental distances and dispersal durations (in bold). (*) Analyses based on main rivers associated with a Strahler number higher than 5 were only performed for lineages occurring in Nigeria.

Path model	Environmental factor	*k*	Regression coefficient	*Q* statistic	p(*Q* > 0)	BF
**Least-cost algorithm**	forests (R)	10	0.051 [0.011, 0.103]	-0.043 [-0.082, -0.013]	0.01	-
		100	0.006 [0.000, 0.043]	-0.080 [-0.144, -0.036]	0.00	-
		1000	0.002 [0.000, 0.036]	-0.083 [-0.146, -0.036]	0.00	-
	forests (C)	10	0.091 [0.047, 0.140]	0.002 [-0.025, 0.020]	0.63	-
		100	0.089 [0.049, 0.129]	-0.001 [-0.041, 0.023]	0.47	-
		1000	0.089 [0.049, 0.129]	-0.001 [-0.043, 0.025]	0.47	-
	grasslands (R)	10	0.091 [0.040, 0.152]	0.001 [-0.001, 0.002]	0.71	-
		100	0.084 [0.044, 0.136]	-0.006 [-0.024, 0.008]	0.17	-
		1000	0.020 [0.010, 0.049]	-0.069 [-0.127, -0.010]	0.01	-
	grasslands (C)	10	0.094 [0.036, 0.169]	0.002 [-0.009, 0.014]	0.63	-
		100	0.092 [0.028, 0.181]	0.001 [-0.019, 0.027]	0.52	-
		1000	0.088 [0.024, 0.176]	-0.004 [-0.023, 0.025]	0.37	-
	savannas (R)	10	0.148 [0.040, 0.264]	0.052 [-0.005, 0.116]	0.95	11.5
		100	0.134 [0.033, 0.226]	0.040 [-0.018, 0.093]	0.93	5.2
		1000	0.130 [0.029, 0.213]	0.030 [-0.024, 0.091]	0.86	-
	savannas (C)	10	0.064 [0.026, 0.115]	-0.024 [-0.080, 0.003]	0.08	-
		100	0.040 [0.016, 0.109]	-0.047 [-0.118, 0.000]	0.03	-
		1000	0.019 [0.007, 0.084]	-0.065 [-0.133, -0.007]	0.02	-
	croplands (R)	10	0.093 [0.049, 0.143]	0.001 [-0.044, 0.038]	0.52	-
		100	0.086 [0.045, 0.134]	-0.003 [-0.071, 0.044]	0.45	-
		1000	0.084 [0.044, 0.131]	-0.005 [-0.076, 0.044]	0.43	-
	croplands (C)	10	0.114 [0.031, 0.183]	0.016 [-0.020, 0.054]	0.71	-
		100	0.081 [0.009, 0.109]	-0.021 [-0.074, 0.032]	0.20	-
		1000	0.070 [0.001, 0.096]	-0.034 [-0.089, 0.026]	0.11	-
	elevation (R)	10	0.145 [0.065, 0.230]	0.055 [0.019, 0.091]	1.00	19.0
		100	0.168 [0.076, 0.269]	0.074 [0.016, 0.136]	1.00	10.1
		1000	0.168 [0.075, 0.272]	0.072 [0.014, 0.132]	0.98	9.0
	elevation (C)	10	0.054 [0.025, 0.096]	-0.037 [-0.062, -0.010]	0.00	-
		100	0.040 [0.020, 0.070]	-0.052 [-0.089, -0.016]	0.00	-

		1000	0.036 [0.018, 0.064]	-0.055 [-0.094, -0.018]	0.00	-
	annual mean temperature (R)	10	0.088 [0.037, 0.148]	-0.003 [-0.007, 0.000]	0.01	-
		100	0.088 [0.037, 0.147]	-0.003 [-0.007, 0.000]	0.01	-
		1000	0.088 [0.037, 0.147]	-0.003 [-0.007, 0.000]	0.01	-
	annual mean temperature (C)	10	0.095 [0.041, 0.161]	0.004 [0.001, 0.008]	1.00	4.3
		100	0.095 [0.041, 0.161]	0.004 [0.001, 0.009]	1.00	4.3
		1000	0.095 [0.041, 0.162]	0.004 [0.001, 0.009]	1.00	4.3
	annual precipitation (R)	10	0.091 [0.033, 0.150]	-0.003 [-0.019, 0.006]	0.27	-
		100	0.089 [0.030, 0.148]	-0.004 [-0.025, 0.006]	0.22	-
		1000	0.089 [0.030, 0.148]	-0.005 [-0.025, 0.007]	0.22	-
	annual precipitation (C)	10	0.088 [0.039, 0.154]	-0.002 [-0.009, 0.014]	0.38	-
		100	0.086 [0.039, 0.153]	-0.003 [-0.012, 0.017]	0.36	-
		1000	0.086 [0.039, 0.152]	-0.003 [-0.012, 0.017]	0.36	-
	human population density (log_{10}, R)	10	0.083 [0.035, 0.152]	-0.006 [-0.016, 0.002]	0.09	-
		100	0.082 [0.035, 0.150]	-0.008 [-0.019, 0.002]	0.07	-
		1000	0.082 [0.035, 0.150]	-0.008 [-0.020, 0.002]	0.06	-
	human population density (log_{10}, C)	10	0.097 [0.042, 0.149]	0.002 [-0.008, 0.010]	0.68	-
		100	0.098 [0.043, 0.150]	0.003 [-0.009, 0.012]	0.71	-
		1000	0.098 [0.043, 0.150]	0.003 [-0.009, 0.012]	0.70	-
	main rivers (*S* > 2, R)	10	0.066 [0.039, 0.117]	-0.002 [-0.006, 0.000]	0.05	-
		100	0.053 [0.032, 0.101]	-0.013 [-0.032, -0.002]	0.01	-
		1000	0.022 [0.012, 0.051]	-0.043 [-0.081, -0.015]	0.00	-
	main rivers (*S* > 3, R)	10	0.066 [0.040, 0.118]	-0.001 [-0.002, 0.001]	0.20	-
		100	0.055 [0.033, 0.106]	-0.010 [-0.022, -0.001]	0.02	-
		1000	0.019 [0.010, 0.045]	-0.047 [-0.092, -0.022]	0.00	-
	main rivers (*S* > 4, R)	10	0.067 [0.040, 0.119]	0.000 [-0.001, 0.001]	0.49	-
		100	0.065 [0.038, 0.114]	-0.002 [-0.010, 0.004]	0.26	-
		1000	0.031 [0.015, 0.062]	-0.034 [-0.076, -0.015]	0.00	-
	main rivers (*S* > 5, R)	10	0.067 [0.040, 0.119]	0.000 [-0.001, 0.001]	0.48	-
		100	0.065 [0.038, 0.114]	-0.002 [-0.010, 0.004]	0.26	-
		1000	0.031 [0.015, 0.062]	-0.034 [-0.076, -0.015]	0.00	-
	main rivers (*S* > 6, R)	10	0.066 [0.040, 0.119]	0.000 [-0.001, 0.000]	0.02	-
		100	0.062 [0.035, 0.114]	-0.003 [-0.01, -0.001]	0.01	-
		1000	0.021 [0.008, 0.043]	-0.045 [-0.079, -0.027]	0.00	-
**Circuitscape algorithm**	forests (R)	10	0.008 [0.001, 0.022]	-0.071 [-0.098, -0.037]	0.00	-
		100	0.001 [0.000, 0.009]	-0.079 [-0.107, -0.038]	0.00	-
		1000	0.001 [0.000, 0.008]	-0.080 [-0.108, -0.038]	0.00	-
	forests (C)	10	0.093 [0.055, 0.127]	0.015 [-0.004, 0.030]	0.95	1.9
		100	0.083 [0.050, 0.117]	0.004 [-0.017, 0.026]	0.73	-
		1000	0.077 [0.047, 0.113]	-0.002 [-0.024, 0.025]	0.44	-
	grasslands (R)	10	0.077 [0.043, 0.107]	-0.001 [-0.010, 0.007]	0.42	-
		100	0.031 [0.018, 0.059]	-0.045 [-0.073, -0.009]	0.01	-
		1000	0.011 [0.004, 0.028]	-0.067 [-0.097, -0.022]	0.00	-
	grasslands (C)	10	0.077 [0.034, 0.103]	-0.004 [-0.008, 0.000]	0.06	-
		100	0.070 [0.028, 0.097]	-0.011 [-0.020, -0.003]	0.00	-
		1000	0.063 [0.024, 0.093]	-0.016 [-0.029, -0.006]	0.00	-
	savannas (R)	10	0.127 [0.057, 0.188]	0.050 [0.009, 0.095]	0.99	13.3
		100	0.121 [0.048, 0.187]	0.038 [-0.008, 0.096]	0.94	**24.0**
		1000	0.116 [0.046, 0.184]	0.034 [-0.012, 0.093]	0.90	19.0
	savannas (C)	10	0.039 [0.017, 0.058]	-0.043 [-0.066, -0.016]	0.00	-
		100	0.028 [0.012, 0.051]	-0.050 [-0.078, -0.018]	0.00	-
		1000	0.028 [0.010, 0.057]	-0.051 [-0.083, -0.011]	0.00	-
	croplands (R)	10	0.060 [0.031, 0.084]	-0.019 [-0.038, 0.004]	0.08	-
		100	0.054 [0.028, 0.079]	-0.025 [-0.047, 0.004]	0.05	-
		1000	0.053 [0.027, 0.078]	-0.026 [-0.049, 0.004]	0.05	-
	croplands (C)	10	0.070 [0.021, 0.105]	-0.010 [-0.037, 0.024]	0.20	-
		100	0.046 [0.001, 0.072]	-0.037 [-0.071, 0.009]	0.05	-
		1000	0.042 [0.001, 0.064]	-0.040 [-0.074, -0.002]	0.03	-
	elevation (R)	10	0.132 [0.064, 0.179]	0.050 [0.024, 0.081]	1.00	10.1
		100	0.149 [0.077, 0.207]	0.066 [0.027, 0.114]	1.00	10.1

		1000	0.150 [0.079, 0.210]	0.067 [0.026, 0.118]	1.00	11.5
	elevation (C)	10	0.039 [0.017, 0.060]	-0.040 [-0.057, -0.019]	0.00	-
		100	0.015 [0.006, 0.029]	-0.063 [-0.085, -0.031]	0.00	-
		1000	0.010 [0.004, 0.021]	-0.068 [-0.091, -0.034]	0.00	-
	annual mean temperature (R)	10	0.076 [0.036, 0.106]	-0.004 [-0.008, -0.001]	0.00	-
		100	0.076 [0.036, 0.105]	-0.004 [-0.008, -0.002]	0.00	-
		1000	0.076 [0.036, 0.105]	-0.004 [-0.008, -0.002]	0.00	-
	annual mean temperature (C)	10	0.083 [0.039, 0.112]	0.003 [0.000, 0.007]	0.99	0.8
		100	0.084 [0.039, 0.112]	0.003 [0.000, 0.008]	0.99	0.8
		1000	0.084 [0.039, 0.112]	0.003 [0.000, 0.008]	0.99	0.8
	annual precipitation (R)	10	0.050 [0.022, 0.071]	-0.030 [-0.040, -0.016]	0.00	-
		100	0.044 [0.018, 0.064]	-0.036 [-0.049, -0.019]	0.00	-
		1000	0.043 [0.018, 0.063]	-0.037 [-0.050, -0.020]	0.00	-
	annual precipitation (C)	10	0.099 [0.049, 0.130]	0.018 [0.009, 0.028]	1.00	1.9
		100	0.103 [0.052, 0.133]	0.021 [0.010, 0.033]	1.00	1.8
		1000	0.103 [0.052, 0.134]	0.021 [0.010, 0.033]	1.00	1.8
	human population density (log$_{10}$, R)	10	0.066 [0.030, 0.094]	-0.012 [-0.019, -0.003]	0.00	-
		100	0.064 [0.029, 0.091]	-0.014 [-0.023, -0.004]	0.00	-
		1000	0.063 [0.028, 0.090]	-0.014 [-0.024, -0.004]	0.00	-
	human population density (log$_{10}$, C)	10	0.093 [0.046, 0.122]	0.009 [0.002, 0.018]	0.99	13.3
		100	0.094 [0.046, 0.123]	0.010 [0.001, 0.022]	0.98	11.5
		1000	0.094 [0.046, 0.123]	0.010 [0.001, 0.022]	0.98	11.5

Table S4. Impact of several environmental factors on the dispersal velocity of Lassa virus tip branches, based on the analysis of segment S. The results are based on 100 posterior trees obtained by spatially-explicit phylogeographic inference. For the main rivers *S* refers to the Strahler number, which measures the importance of a river by counting the number of upstream rivers connected to it. "C" and "R" indicate if the considered environmental raster was considered as a conductance ("C") or resistance factor ("R"), "*k*" is the rescaling parameter used to transform the initial raster, and "*S*" refers to the Strahler number that measures its importance by looking at the number of upstream rivers connected to it (see the text for further detail). For regression coefficients and *Q* values we report both the median estimate and the 95% HPD interval. The Bayes factor (BF) supports are only reported when p(*Q* > 0) is at least 90%. Following Kass & Raftery (1995), we consider a BF value >20 as strong support for a significant correlation between the environmental distances and dispersal durations (in bold). (*) Analyses based on main rivers associated with a Strahler number higher than 5 were only performed for lineages occurring in Nigeria.

Path model	Environmental factor	*k*	Regression coefficient	*Q* statistic	p(*Q* > 0)	BF
**Least-cost algorithm**	forests (R)	10	0.033 [0.017, 0.054]	-0.034 [-0.051, -0.018]	0.00	-
		100	0.002 [0.000, 0.010]	-0.063 [-0.085, -0.044]	0.00	-
		1000	0.001 [0.000, 0.006]	-0.065 [-0.086, -0.046]	0.00	-
	forests (C)	10	0.083 [0.061, 0.112]	0.018 [0.008, 0.027]	1.00	1.9
		100	0.087 [0.063, 0.118]	0.021 [0.010, 0.035]	1.00	1.3
		1000	0.088 [0.065, 0.120]	0.023 [0.011, 0.037]	1.00	1.3
	grasslands (R)	10	0.068 [0.050, 0.088]	0.001 [0.000, 0.003]	0.97	5.7
		100	0.068 [0.049, 0.095]	0.002 [-0.007, 0.019]	0.62	-
		1000	0.030 [0.019, 0.077]	-0.035 [-0.056, 0.005]	0.06	-
	grasslands (C)	10	0.068 [0.044, 0.093]	0.001 [-0.007, 0.011]	0.54	-
		100	0.057 [0.034, 0.083]	-0.010 [-0.020, 0.006]	0.10	-
		1000	0.049 [0.028, 0.074]	-0.019 [-0.029, -0.001]	0.02	-
	savannas (R)	10	0.078 [0.055, 0.098]	0.010 [-0.007, 0.033]	0.90	11.5
		100	0.051 [0.030, 0.083]	-0.014 [-0.040, 0.026]	0.11	-
		1000	0.038 [0.017, 0.066]	-0.028 [-0.056, 0.013]	0.04	-
	savannas (C)	10	0.078 [0.049, 0.111]	0.011 [-0.004, 0.029]	0.90	1.0
		100	0.071 [0.044, 0.114]	0.007 [-0.016, 0.040]	0.67	-
		1000	0.068 [0.044, 0.110]	0.005 [-0.024, 0.039]	0.57	-
	croplands (R)	10	0.109 [0.068, 0.147]	0.040 [0.019, 0.061]	1.00	1.9
		100	0.123 [0.078, 0.168]	0.053 [0.026, 0.082]	1.00	2.0
		1000	0.125 [0.079, 0.171]	0.055 [0.026, 0.085]	1.00	1.9
	croplands (C)	10	0.060 [0.040, 0.080]	-0.007 [-0.026, 0.010]	0.16	-
		100	0.017 [0.006, 0.034]	-0.048 [-0.072, -0.028]	0.00	-
		1000	0.003 [0.000, 0.012]	-0.062 [-0.085, -0.046]	0.00	-
	elevation (R)	10	0.098 [0.066, 0.137]	0.031 [0.015, 0.054]	1.00	1.9

		100	0.112 [0.069, 0.169]	0.048 [0.020, 0.086]	1.00	1.4
		1000	0.113 [0.068, 0.172]	0.050 [0.020, 0.090]	1.00	1.4
	elevation (C)	10	0.046 [0.035, 0.058]	-0.020 [-0.032, -0.011]	0.00	-
		100	0.034 [0.025, 0.044]	-0.031 [-0.047, -0.018]	0.00	-
		1000	0.027 [0.019, 0.036]	-0.038 [-0.054, -0.024]	0.00	-
	annual mean temperature (R)	10	0.065 [0.048, 0.084]	-0.001 [-0.004, 0.000]	0.07	-
		100	0.065 [0.048, 0.083]	-0.001 [-0.004, 0.000]	0.07	-
		1000	0.065 [0.048, 0.083]	-0.001 [-0.005, 0.000]	0.07	-
	annual mean temperature (C)	10	0.068 [0.049, 0.089]	0.002 [0.000, 0.004]	0.98	0.4
		100	0.068 [0.049, 0.089]	0.002 [0.000, 0.005]	0.98	0.4
		1000	0.068 [0.049, 0.089]	0.002 [0.000, 0.005]	0.98	0.4
	annual precipitation (R)	10	0.056 [0.040, 0.070]	-0.010 [-0.017, -0.005]	0.00	-
		100	0.053 [0.038, 0.066]	-0.013 [-0.022, -0.007]	0.00	-
		1000	0.053 [0.038, 0.066]	-0.013 [-0.023, -0.007]	0.00	-
	annual precipitation (C)	10	0.073 [0.052, 0.097]	0.007 [0.003, 0.012]	1.00	1.5
		100	0.074 [0.053, 0.100]	0.008 [0.004, 0.015]	1.00	1.4
		1000	0.074 [0.053, 0.100]	0.008 [0.004, 0.015]	1.00	1.4
	human population density ($\log_{10}$, R)	10	0.064 [0.046, 0.085]	-0.002 [-0.005, 0.002]	0.08	-
		100	0.063 [0.046, 0.085]	-0.002 [-0.006, 0.002]	0.09	-
		1000	0.063 [0.046, 0.085]	-0.002 [-0.006, 0.002]	0.09	-
	human population density ($\log_{10}$, C)	10	0.069 [0.051, 0.090]	0.003 [0.000, 0.007]	0.99	3.0
		100	0.070 [0.052, 0.091]	0.004 [0.000, 0.008]	0.99	3.2
		1000	0.070 [0.052, 0.091]	0.004 [0.001, 0.008]	0.99	3.2
	main rivers (S > 2, R)	10	0.077 [0.053, 0.114]	0.000 [-0.002, 0.002]	0.57	-
		100	0.068 [0.047, 0.101]	-0.008 [-0.022, 0.002]	0.06	-
		1000	0.038 [0.026, 0.057]	-0.038 [-0.070, -0.017]	0.00	-
	main rivers (S > 3, R)	10	0.076 [0.053, 0.112]	-0.001 [-0.002, 0.000]	0.16	-
		100	0.068 [0.048, 0.101]	-0.008 [-0.019, 0.001]	0.04	-
		1000	0.033 [0.021, 0.056]	-0.044 [-0.074, -0.021]	0.00	-
	main rivers (S > 4, R)	10	0.076 [0.053, 0.113]	0.000 [-0.001, 0.000]	0.31	-
		100	0.073 [0.050, 0.105]	-0.003 [-0.011, 0.002]	0.10	-
		1000	0.036 [0.024, 0.048]	-0.039 [-0.069, -0.020]	0.00	-
	main rivers (S > 5, R)	10	0.076 [0.053, 0.113]	0.000 [-0.001, 0.000]	0.30	-
		100	0.073 [0.050, 0.105]	-0.003 [-0.011, 0.001]	0.09	-
		1000	0.036 [0.024, 0.048]	-0.040 [-0.069, -0.020]	0.00	-
	main rivers (S > 6, R)	10	0.076 [0.052, 0.112]	-0.001 [-0.001, 0.000]	0.02	-
		100	0.069 [0.046, 0.102]	-0.007 [-0.013, -0.003]	0.01	-
		1000	0.020 [0.011, 0.031]	-0.057 [-0.089, -0.037]	0.00	-
**Circuitscape algorithm**	forests (R)	10	0.008 [0.001, 0.016]	-0.068 [-0.085, -0.050]	0.00	-
		100	0.001 [0.000, 0.004]	-0.075 [-0.096, -0.056]	0.00	-
		1000	0.001 [0.000, 0.003]	-0.075 [-0.097, -0.056]	0.00	-
	forests (C)	10	0.103 [0.079, 0.127]	0.027 [0.018, 0.042]	1.00	3.5
		100	0.114 [0.089, 0.143]	0.037 [0.027, 0.057]	1.00	4.3
		1000	0.119 [0.094, 0.151]	0.043 [0.033, 0.065]	1.00	5.2
	grasslands (R)	10	0.077 [0.058, 0.102]	0.000 [-0.005, 0.015]	0.54	-
		100	0.039 [0.024, 0.083]	-0.036 [-0.059, 0.004]	0.05	-
		1000	0.018 [0.008, 0.060]	-0.056 [-0.083, -0.019]	0.01	-
	grasslands (C)	10	0.070 [0.053, 0.093]	-0.006 [-0.013, -0.002]	0.00	-
		100	0.056 [0.038, 0.081]	-0.019 [-0.030, -0.010]	0.00	-
		1000	0.046 [0.029, 0.071]	-0.028 [-0.042, -0.019]	0.00	-
	savannas (R)	10	0.042 [0.028, 0.063]	-0.034 [-0.048, -0.013]	0.00	-
		100	0.020 [0.011, 0.037]	-0.055 [-0.074, -0.031]	0.00	-
		1000	0.016 [0.009, 0.032]	-0.059 [-0.078, -0.035]	0.00	-
	savannas (C)	10	0.060 [0.039, 0.078]	-0.017 [-0.031, -0.007]	0.00	-
		100	0.055 [0.034, 0.078]	-0.020 [-0.044, -0.001]	0.03	-
		1000	0.058 [0.033, 0.089]	-0.015 [-0.048, 0.011]	0.16	-
	croplands (R)	10	0.092 [0.062, 0.116]	0.016 [0.002, 0.027]	0.98	0.8
		100	0.092 [0.060, 0.117]	0.016 [0.000, 0.030]	0.97	1.3
		1000	0.092 [0.060, 0.118]	0.016 [0.000, 0.030]	0.95	1.3
	croplands (C)	10	0.018 [0.007, 0.036]	-0.057 [-0.073, -0.041]	0.00	-

	100	0.001 [0.000, 0.008]	-0.075 [-0.096, -0.055]	0.00	-
	1000	0.000 [0.000, 0.004]	-0.075 [-0.098, -0.057]	0.00	-
elevation (R)	10	0.112 [0.082, 0.148]	0.036 [0.016, 0.059]	1.00	1.0
	100	0.115 [0.075, 0.161]	0.037 [0.010, 0.070]	1.00	1.1
	1000	0.114 [0.073, 0.161]	0.036 [0.008, 0.070]	1.00	1.2
elevation (C)	10	0.040 [0.028, 0.054]	-0.035 [-0.049, -0.025]	0.00	-
	100	0.015 [0.009, 0.023]	-0.061 [-0.081, -0.045]	0.00	-
	1000	0.006 [0.003, 0.011]	-0.070 [-0.093, -0.053]	0.00	-
annual mean temperature (R)	10	0.074 [0.056, 0.097]	-0.001 [-0.005, 0.001]	0.09	-
	100	0.074 [0.056, 0.097]	-0.002 [-0.005, 0.001]	0.07	-
	1000	0.074 [0.056, 0.097]	-0.002 [-0.006, 0.001]	0.07	-
annual mean temperature (C)	10	0.079 [0.059, 0.103]	0.003 [-0.001, 0.006]	0.96	0.2
	100	0.079 [0.059, 0.104]	0.004 [0.000, 0.007]	0.97	0.2
	1000	0.080 [0.059, 0.104]	0.004 [0.000, 0.007]	0.97	0.2
annual precipitation (R)	10	0.045 [0.030, 0.063]	-0.030 [-0.038, -0.023]	0.00	-
	100	0.039 [0.025, 0.055]	-0.037 [-0.046, -0.028]	0.00	-
	1000	0.038 [0.024, 0.054]	-0.038 [-0.047, -0.029]	0.00	-
annual precipitation (C)	10	0.100 [0.075, 0.126]	0.023 [0.017, 0.032]	1.00	2.3
	100	0.103 [0.079, 0.129]	0.027 [0.018, 0.037]	1.00	2.3
	1000	0.104 [0.079, 0.130]	0.027 [0.018, 0.038]	1.00	2.3
human population density (log_{10}, R)	10	0.064 [0.048, 0.086]	-0.011 [-0.016, -0.005]	0.00	-
	100	0.062 [0.046, 0.083]	-0.013 [-0.019, -0.006]	0.00	-
	1000	0.062 [0.046, 0.083]	-0.014 [-0.019, -0.006]	0.00	-
human population density (log_{10}, C)	10	0.083 [0.064, 0.108]	0.007 [0.001, 0.012]	1.00	11.5
	100	0.083 [0.064, 0.107]	0.007 [0.000, 0.013]	0.98	6.7
	1000	0.083 [0.064, 0.107]	0.007 [0.000, 0.013]	0.97	6.1

Table S5. Online platforms used to retrieve sampling coordinates for Lassa virus samples.

Original dataset	URL
OpenStreetMap	https://data.humdata.org/dataset/open-street-map-data-on-guinea-liberia-and-sierra-leone
GeoNames gazetteers	https://www.geonames.org
UN-OCHA (for Sierra Leone)	https://data.humdata.org/dataset/sierra-leone-settlements
UN-OCHA (for Liberia)	https://data.humdata.org/dataset/liberia-settlements-0
Who's on First gazetteer	https://www.whosonfirst.org/docs
Google geocoding API	https://developers.google.com/maps/documentation/geocoding/overview

Table S6. Source of data for each environmental raster.

Original raster	Source	URL
Elevation raster	SRTM (Shuttle Radar Topography Mission)	webmap.ornl.gov
Land cover raster	IGBP (International Geosphere Biosphere Programme) – categorical raster	www.igbp.net
Annual mean temperature	WorldClim database, version 2.0 (bioclimatic variable “bio1”)	worldclim.org
Annual precipitation	WorldClim database, version 2.0 (bioclimatic variable “bio12”)	worldclim.org
Rivers of Africa	Food and Agriculture Organization of the United Nations	data.apps.fao.org
