# Supplementary material for: Predicting the evolution of the Lassa virus endemic area and population at risk over the next decades
Source: Nat Commun. 2022 Sep 27;13:5596. doi: 10.1038/s41467-022-33112-3 (PMC9515147; doi:10.1038/s41467-022-33112-3)
Supplement: Supplementary file 3 — Reporting Summary [file 41467_2022_33112_MOESM3_ESM.pdf]

nature portfolio

Corresponding author(s): Raphaelle Klitting

Last updated by author(s): 8/15/2022

Reporting Summary

Nature Portfolio wishes to improve the reproducibility of the work that we publish. This form provides structure for consistency and transparency in reporting. For further information on Nature Portfolio policies, see our Editorial Policies and the Editorial Policy Checklist.

Statistics

For all statistical analyses, confirm that the following items are present in the figure legend, table legend, main text, or Methods section.

n/a	Confirmed	
☐	☒	The exact sample size (*n*) for each experimental group/condition, given as a discrete number and unit of measurement
☒	☐	A statement on whether measurements were taken from distinct samples or whether the same sample was measured repeatedly
☐	☒	The statistical test(s) used AND whether they are one- or two-sided<br>*Only common tests should be described solely by name; describe more complex techniques in the Methods section.*
☐	☒	A description of all covariates tested
☐	☒	A description of any assumptions or corrections, such as tests of normality and adjustment for multiple comparisons
☐	☒	A full description of the statistical parameters including central tendency (e.g. means) or other basic estimates (e.g. regression coefficient) AND variation (e.g. standard deviation) or associated estimates of uncertainty (e.g. confidence intervals)
☐	☒	For null hypothesis testing, the test statistic (e.g. *F*, *t*, *r*) with confidence intervals, effect sizes, degrees of freedom and *P* value noted<br>*Give P values as exact values whenever suitable.*
☐	☒	For Bayesian analysis, information on the choice of priors and Markov chain Monte Carlo settings
☒	☐	For hierarchical and complex designs, identification of the appropriate level for tests and full reporting of outcomes
☒	☐	Estimates of effect sizes (e.g. Cohen's *d*, Pearson's *r*), indicating how they were calculated

Our web collection on statistics for biologists contains articles on many of the points above.

Software and code

Policy information about availability of computer code

Data collection	No software was used for data collection.
Data analysis	For the ecological niche modelling analyses, we employed the boosted regression trees approach implemented in the R package “dismo” (version 1.3-8) and selected the optimal number of trees for the BRT models using a spatial cross-validation procedure based on five spatially separated folds generated with the “blockCV” R package (version 2.1.4). The alignment of genomic sequences was made using the program MAFFT version 7 (https://mafft.cbrc.jp/alignment/server/) and manually edited using the program AliView version 1.26 (https://ormbunkar.se/aliview/). Continuous phylogeographic and phylodynamic inferences were performed with the Bayesian methods implemented in the open-source program BEAST version 1.10.4 (https://beast.community/programs) and the BEAGLE library (version 3) to improve computational performance (https://github.com/beagle-dev/beagle-lib). We used the program Tracer version 1.7 (https://beast.community/tracer) to identify the number of sampled trees to discard as burn-in as well as to inspect the convergence and mixing properties of the BEAST outputs. We used the program TreeAnnotator version 1.10.4 (https://beast.community/programs) to obtain the maximum clade credibility tree. Subsequent dispersal statistics estimation and landscape phylogeographic analyses were implemented and performed with R functions available in the open-source package "seraphim" version 1.0 (https://github.com/sdellicour/seraphim). The BEAST XML files of the continuous phylogeographic and skygrid analyses, as well as the R scripts and related files needed to run all the ecological niche modelling analyses, landscape phylogeographic testing analyses, and phylogeographic simulations, are available at https://github.com/sdellicour/lassa_spreads.

For manuscripts utilizing custom algorithms or software that are central to the research but not yet described in published literature, software must be made available to editors and reviewers. We strongly encourage code deposition in a community repository (e.g. GitHub). See the Nature Portfolio guidelines for submitting code & software for further information.

Data

Policy information about availability of data

All manuscripts must include a data availability statement. This statement should provide the following information, where applicable:

- Accession codes, unique identifiers, or web links for publicly available datasets
- A description of any restrictions on data availability
- For clinical datasets or third party data, please ensure that the statement adheres to our policy

All source data used in this study are available at https://github.com/sdellicour/lassa_spreads (DOI: 10.5281/zenodo.6998624). The sources of the different raster files used in this study are provided in Table S6. We obtained occurrence data for the Muridae family from the Global Biodiversity Information Facility (http://www.gbif.org, accessed 2019-07-19, GBIF occurrence downloads https://doi.org/10.15468/dl.cs3c41). For the M. natalensis species, we obtained occurrence data from the Global Biodiversity Information Facility (http://www.gbif.org, accessed 2019-07-19, GBIF occurrence downloads https://doi.org/10.15468/dl.hrjyj1), the Integrated Digitized Biocollections (http://www.idigbio.org/portal (2020), Query: {"filtered": {"filter": {"and": [{"exists": {"field": "geopoint"}}, {"term": {"scientificname": "mastomys natalensis"}}]}}}, 4348 records, accessed on 2020-01-04T05:40:40.066945, contributed by 19 Recordsets), the Field Museum of Natural History Zoological collections (Field Museum of Natural History (Zoology) Mammal Collection https://collections-zoology.fieldmuseum.org/list?f%5B0%5D=ss_CatCatalog%3A%22Mammals%22&_ga=2.123662347.1070684726.1508778418-143671043.1493067972, accessed 2019-12-13), and the African Mammalia database (African Mammalia, http://projects.biodiversity.be/africanmammalia/search, accessed 2019-12-14). Full citations are provided in "Citations_rodent_occurrence_data.txt" available on the GitHub repository referenced above. This data set was supplemented with the data available in the scientific literature (search for term "Mastomys natalensis", in PubMed and Google). For each record used in this analysis, the specific record or collection ID is specified in the file "Mastomys_natalensis_RK050820.csv" or "Muridae_family_allData_RK220819.csv", both available on the GitHub repository referenced above. The sources used to retrieve sampling coordinates for Lassa virus samples are listed in Table S5. For Lassa virus, occurrence data were obtained from the scientific literature (search for term "Lassa virus", in PubMed and Google) and the source of each record used in this analysis is specified in the file "Lassa_virus_cases_RK070820.csv" available on the GitHub repository referenced above. The sources of the different raster files used in this study are provided in Table S6. Data for the environmental factors used in the BRT analyses was obtained from the Inter-Sectoral Impact Model Intercomparison Project phase 2b (ISIMIP2b, https://data.isimip.org/). LASV sequences analysed in the present study were available on GenBank before November 20, 2019, except for the LASV sequences from cases sampled during the year 2019 in Nigeria, which are are publicly available on the website virological.org (https://virological.org/t/2019-lassa-virus-sequencing-in-nigeria-final-field-report-75-samples/291). Accession numbers of selected genomic sequences are listed in the file "LASV_all_the_metadata.csv" available on the GitHub repository referenced above. All processed data (BRT models, BRT predictions, phylogeographic inferences, dispersal statistics estimations, and seraphim analyses) generated in this study are also available on the GitHub repository referenced above.

Field-specific reporting

Please select the one below that is the best fit for your research. If you are not sure, read the appropriate sections before making your selection.

- [ ] Life sciences
- [ ] Behavioural & social sciences
- [x] Ecological, evolutionary & environmental sciences

For a reference copy of the document with all sections, see nature.com/documents/nr-reporting-summary-flat.pdf

Ecological, evolutionary & environmental sciences study design

All studies must disclose on these points even when the disclosure is negative.

Study description	In our study we performed ecological niche modelling and phylogeographic analyses to model how the endemic range of Lassa virus (LASV) may evolve in the next five decades in response to climate change, human population growth, and land use changes. Specifically, (i) we performed ecological niche modelling analyses for Lassa virus and his reservoir host, Mastomys natalensis to identify the determinants of ecological suitability for LASV, (ii) we projected the future ecological suitability for LASV across Africa, (iii) we estimated the human population living in areas suitable for LASV based on human population projections, (iv) we used a continuous phylogeographic approach to reconstruct the dispersal history of LASV in West Africa and estimate the virus mean lineage dispersal velocity, (v) we used a first landscape phylogeographic approach to test the impact of main waterways on the dispersal history of LASV lineages, (vi) we used a second landscape phylogeographic approach to test the impact of environmental factors on the dispersal velocity of LASV lineages, and (vii) we used phylogeographic simulations to illustrate how a slow lineage dispersal velocity may limit the spatial extent of LASV spread following a potential introduction event.
Research sample	The rodent data sets used in the BRT analyses consist in 2,504 Mastomys natalensis spatial occurrence records and in 10,806 Muridae occurrence records. The M. natalensis dataset is meant to represent the locations where this rodent species is present. The Muridae dataset is meant to serve for sampling pseudo-absences and represents locations where the Muridae family is present. We obtained occurrence data for the Muridae family from the Global Biodiversity Information Facility (http://www.gbif.org, accessed 2019-07-19, GBIF occurrence downloads https://doi.org/10.15468/dl.cs3c41. We obtained occurrence data for the M. natalensis species from the Global Biodiversity Information Facility (http://www.gbif.org, accessed 2019-07-19, GBIF occurrence downloads https://doi.org/10.15468/dl.hrjyj1), the Integrated Digitized Biocollections (http://www.idigbio.org/portal (2020), Query: {"filtered": {"filter": {"and": [{"exists": {"field": "geopoint"}}, {"term": {"scientificname": "mastomys natalensis"}}]}}}, 4348 records, accessed on 2020-01-04T05:40:40.066945, contributed by 19 Recordsets), the Field Museum of Natural History Zoological collections (Field Museum of Natural History (Zoology) Mammal Collection https://collections-zoology.fieldmuseum.org/list?f%5B0%5D=ss_CatCatalog%3A%22Mammals%22&_ga=2.123662347.1070684726.1508778418-143671043.1493067972, accessed 2019-12-13), and the African Mammalia database (African Mammalia, http://projects.biodiversity.be/africanmammalia/search, accessed 2019-12-14).<br>The BRT analyses were based on several environmental factors: harmonised present-day and future climate, land cover and population data available through the Inter-Sectoral Impact Model Intercomparison Project phase 2b (ISIMIP2b, https://data.isimip.org/). They are meant to represent the mean values observed over a spatial grid at a 0.5 decimal degrees resolution throughout Africa for the environmental factors stated above at present-time (1986-2005) and projected over the next decades

	(2021-2040, 2041-2060, and 2061-2080) according to three different climate scenario (SSP1-26, SSP4-6.0 and SSP8-85). The analysed LASV sequence data sets consist in 756 S segment sequences and 551 L segment sequences sampled in West Africa, they are meant to represent the most complete set of publicly available Lassa virus genomes that (1) have been identified in natural settings (in rodents or humans), and (2), for which the collection time is known.
Sampling strategy	The climate information consists of daily gridded near-surface air temperature and surface precipitation fields derived from four bias-adjusted global climate models (GCMs; GFDL-ESM2M, HadGEM2-ES, IPSL-CM5A-LR, and MIROC5) participating in the fifth phase of the Coupled Model Intercomparison Project (CMIP5). We considered simulations conducted under historical climate forcings and RCPs 2.6, 6.0 and 8.5. In addition, we considered observed gridded temperature and precipitation from the concatenated products GSWP3 and EWEMBI for assessing the current (1986-2005) conditions. For land cover we use version 2 of the Land Use Harmonisation (LUH2) providing historical and projected land cover states under a range of shared socioeconomic pathways (SSPs), and from which we consider SSP1-26, SSP4-6.0 and SSP8-85. Finally, we retrieve gridded population projections under SSP2-26. For each combination of product (GCM, GSWP3-EWEMBI LUH2, gridded population), scenario (historical, RCP, SSP) and analysis window (1986-2005, 2021-2040, 2041-2060, and 2061-2080), we compute the grid-scale temporal mean. For both Mastomys natalensis and Muridae rodents data sets, duplicate occurrence records as well as occurrence records located in the ocean were excluded from the final rodent occurrence data sets (Mastomys natalensis and Muridae). For this study, we retrieved all Lassa virus sequences available on GenBank (November 20, 2019) and we also included in our data sets new sequences generated in 2019 and not available on GenBank (https://virological.org/t/2019-lassa-virus-sequencing-in-nigeria-final-field-report-75-samples/291). We then filtered the sequence data by: (i) excluding laboratory strains (adapted, passaged multiple times, recombinant, obtained from antiviral or vaccine experiments), (ii) excluding sequences without a timestamp, (iii) keeping only sequences from a single time point (if multiple time points were available for a patient), (iv) removing duplicates (when more than one sequence was available for a single strain), and (v) excluding sequences from identified hospital epidemics or sequences for which the location corresponded to the site of hospitalisation. Occurrence and sequence data correspond to all the available data we managed to collect and retrieve at the time of the study. The resulting data sets cover the respective ranges of the rodent host and Lassa virus.
Data collection	We obtained data for the environmental factors used in the BRT analyses from the Inter-Sectoral Impact Model Intercomparison Project phase 2b (ISIMIP2b, https://data.isimip.org/). We collected M. natalensis species occurrence data online from publicly available databases and museum collections: from the Global Biodiversity Information Facility (http:// www.gbif.org, accessed 2019-07-19, GBIF Occurrence Download: https://doi.org/10.15468/dl.hrjyj1), the Integrated Digitized Biocollections (http:// www.idigbio.org/portal (2020), Query: {"filtered": {"filter": {"and": [{"exists": {"field": "geopoint"}}, {"term": {"scientificname": "mastomys natalensis"}}]}}}, 4348 records, accessed on 2020-01-04T05:40:40.066945, contributed by 19 Recordsets), the Field Museum of Natural History Zoological collections (Field Museum of Natural History (Zoology) Mammal Collection https://collections-zoology.fieldmuseum.org/list?f%5B0%5D=ss_CatCatalog%3A%22Mammals%22&_ga=2.123662347.1070684726.1508778418-143671043.1493067972, accessed 2019-12-13), and the African Mammalia database (African Mammalia, http://projects.biodiversity.be/africanmammalia/search, accessed 2019-12-14). This data set was supplemented with the data available in the scientific literature (search for term "Mastomys natalensis", in PubMed and Google). We collected occurrence data for the Muridae family online from the public GBIF database ((http:// www.gbif.org, accessed 2019-07-19, GBIF Occurrence Download: https://doi.org/10.15468/dl.cs3c41).<br>We collected all LASV sequences available online on Nov 20, 2019 on the NCBI Nucleotide database (keywords: "lassa NOT mopeia NOT natalensis"; n = 729 L and 1202 S sequences; database accessed on October 31, 2019) and combining it with sequence data sampled during 2019 in Nigeria (available at https://virological.org/t/2019-lassa-virus-sequencing-in-nigeria-final-field-report-75-samples/291).
Timing and spatial scale	We used environmental data for the African continent and the following analysis windows: analysis window (1986-2005, 2021-2040, 2041-2060, and 2061-2080). The choice of the different time windows was arbitrary but they were defined to cover distinct periods of time. Mastomys natalensis and Muridae occurrence data were collected in Africa up to 2019 and are meant to represent all known spatial records of M. natalensis and Muridae available at the time of the analysis. Genomic sequences for LASV were collected in West Africa between 1969 and 2019 and are meant to represent all Lassa virus genomes publicly available at the time of the analysis that (1) have been identified in natural settings (in rodents or humans), and (2), for which the collection time is known.
Data exclusions	There was no data exclusion.
Reproducibility	(No experiment was performed)
Randomization	There was no group allocation performed in our study.
Blinding	This study is not a clinical research study and does not involve human subjects thus blinding was not necessary.

Did the study involve field work? ☐ Yes ☒ No

Reporting for specific materials, systems and methods

We require information from authors about some types of materials, experimental systems and methods used in many studies. Here, indicate whether each material, system or method listed is relevant to your study. If you are not sure if a list item applies to your research, read the appropriate section before selecting a response.

Materials & experimental systems

n/a	Involved in the study
☒	☐ Antibodies
☒	☐ Eukaryotic cell lines
☒	☐ Palaeontology and archaeology
☒	☐ Animals and other organisms
☒	☐ Human research participants
☒	☐ Clinical data
☒	☐ Dual use research of concern

Methods

n/a	Involved in the study
☒	☐ ChIP-seq
☒	☐ Flow cytometry
☒	☐ MRI-based neuroimaging
